# Complete Genome Sequence of Human Respiratory Syncytial Virus Isolated in Mexico

**DOI:** 10.1128/genomeA.01542-15

**Published:** 2016-01-14

**Authors:** J. E. Muñoz-Medina, I. E. Monroy-Muñoz, A. Santos Coy-Arechavaleta, A. Meza-Chávez, J. Ángeles-Martínez, Y. M. Anguiano-Hernández, C. E. Santacruz-Tinoco, J. González-Ibarra, B. Martínez-Miguel, J. E. Alvarado-Yaah, I. D. Palomec-Nava, J. M. Ortiz-Alcántara, F. Garcés-Ayala, J. E. Ramírez-González, J. A. Díaz-Quiñonez, C. R. González-Bonilla

**Affiliations:** a Laboratorio Central de Epidemiología, Instituto Mexicano del Seguro, Social; b Laboratorio de Genómica, Instituto Nacional de Perinatología, Social; c Hospital de Cardiología CMN SXXI, Instituto Mexicano del Seguro, Social; d Laboratorio de Genómica, Instituto Nacional de Cardiología, Social; e División de Laboratorios de Vigilancia Epidemiológica, Instituto Mexicano del Seguro, Social; f Instituto de Diagnóstico y Referencia Epidemiológicos, Secretaría de Salud; g Facultad de Medicina, Universidad Nacional Autónoma de México

## Abstract

Human respiratory syncytial virus (HRSV) is a member of the *Paramyxoviridae* family, which causes lower respiratory tract infections in neonates and children younger than 5 years. Here, we report the complete genome sequence of HRSV, isolated from a nasopharyngeal swab of a pregnant woman with cardiac complications.

## GENOME ANNOUNCEMENT

Human respiratory syncytial virus (HRSV) is the leading cause of lower respiratory tract infections in neonates and children younger than 5 years (1). These infections can also occur in immunosuppressed and elderly people (2).

This virus belongs to the *Pneumovirus* genus within the *Paramyxoviridae* family (3, 4). The viral genome consists of a linear single-stranded, negative-sense, nonsegmented RNA of about 15.2 kb, which encodes three proteins associated with the nucleocapsid of the nucleoprotein (N), the phosphoprotein (P), and the polymerase or large protein (L), as well as the other five viral proteins found in the viral envelope, including the nonglycosylated matrix proteins (M), the viral membrane proteins (M2-1 and M2-2), the fusion protein (F), the glycoprotein (G), and the short hydrophobic protein (SH). There is only one serotype of HRSV with two major antigenic subgroups—A and B—according to the genetic and antigenic variability of gene G. The strains of both subtypes cocirculate, and although the circulation patterns are not clear, repeated infections and disease severity are related to different subgroups of HRSV (5, 6).

The follow-up and reporting of atypical course cases strengthens further observations. Moreover, obtaining the complete genome of these cases helps to determine viral sequence patterns that may be associated with the development of certain complications.

Nowadays there are few reports that associate the presence of HRSV with cardiac complications (7, 8), and none has obtained the complete genome of the virus.

Here, we report the complete sequence of the genome of HRSV strain 1856 isolated from a nasopharyngeal swab of a 30-year-old pregnant woman at 34.5 weeks of gestation, who started with symptoms of influenzalike illness (ILI) that evolved to severe acute respiratory infection (SARI) and finally presented with cardiogenic shock and myocarditis. The pregnancy was resolved via abdominal surgery and the infant was observed to be healthy.

The patient was treated at the Cardiology Highly Specialized Medical Unit (UMAE) of the National Medical Center Century XXI in Mexico City.

The presence of HRSV was confirmed by reverse transcription (RT)-PCR 8 days after the beginning of the symptoms.

Total RNA was extracted using QIAmp viral RNA minikit (Qiagen, Germany), and 11 primer pairs were used to generate overlapping amplicons that covered the entire viral genome using a SuperScript III One-Step RT-PCR System with Platinum Taq DNA (Invitrogen).

The HRSV strain 1856 was sequenced using the Ion Torrent platform. A single-end library was generated, resulting in 143,843 reads with an average length of 153 bp (22,064,342 bases). The whole-genome sequence of 15,276 nucleotides was assembled with TMAP version 4.2.18 using the Netherlands strain 03-034613 (accession no. JX576759.1) as a mapping reference sequence. The average coverage was 1,321× and only one contig was obtained, annotated, and submitted using the NCBI BankIt tool. The sequence contains the 5′ and 3′ untranslated regions and 11 open reading frames that encode NS1, NS2, N, P, M, SH, G, F, M2-1, M2-2, and L proteins.

### Nucleotide sequence accession number.

The whole-genome sequence of strain 1856 has been deposited in GenBank under the accession number KR350475.
